# Asymmetries in paddle force influence choice of stroke type for canoe slalom athletes 

**DOI:** 10.3389/fphys.2023.1227871

**Published:** 2023-11-21

**Authors:** James M. Wakeling, Stanislava Smiešková, Jaylene S. Pratt, Matej Vajda, Jan Busta

**Affiliations:** ^1^ Department of Biomedical Physiology and Kinesiology, Simon Fraser University, Burnaby, BC, Canada; ^2^ Hamar Institute for Human Performance, Faculty of Physical Education and Sports, Comenius University, Bratislava, Slovakia; ^3^ Faculty of Physical Education and Sport, Charles University, Prague, Czecia

**Keywords:** canoe, kayak, slalom canoe racing, force, power, paddle, stroke

## Abstract

Canoe slalom is an Olympic discipline where athletes race down a whitewater course in kayaks (K1) or canoes (C1) navigating a set of down-stream and up-stream gates. Kayak paddles are symmetrical and have a blade at each end, whereas C1 paddles have only one blade that must be moved across the boat to perform strokes on either the right or left side. Asymmetries in paddle force between the two sides of the boat may lead to a reduction in predicted race time. The purpose of this study was to quantify asymmetries in the paddle forces between the two sides for slalom paddling. Paddle forces for 42 canoe slalom athletes (C1 and K1) were quantified from the straight sections of a flat-water figure-of-eight course. Paddle forces were measured using strain gauges embedded in the paddle shaft, stroke type was identified using video, and boat trajectory was tracked using inertial measurement units and high-speed GPS: data were fused using in-house analysis software. Paddle forces were quantified by their peak force, and impulse during the stroke. Paddle forces for the kayakers had asymmetries of 14.2 to 17.1% for the male K1M and 11.1 to 14.4% for the women K1W. Canoeists were no more asymmetrical than the kayakers for their ‘on-side’ strokes between the right and left sides. However, there were considerable differences for their ‘off-side’ strokes: male C1M off-side paddle forces were similar to their ‘on-side’ forces for the same side, but the women C1W had a significantly lower (−20.8% to −29.5%) paddle forces for their ‘off-side’ strokes compared to their ‘on-side’ strokes on that same side. Despite an increasing number of younger male athletes being introduced to the switching technique, and it being used by C1M athletes in international competitions since 2014, C1M paddlers still do not use switching transitions as much as C1W. The data from this study indicate that there is a biomechanical reason for this sex-based difference in the higher proportion of off-side strokes used by the C1M athletes compared to C1W athletes: and this needs to be considered for optimal technique development and race performance.

## Introduction

Canoe slalom is an Olympic discipline where athletes navigate a set of slalom gates on a whitewater course in the shortest time possible (while incurring time penalties for touching or missing the gates). There are between 18 and 25 gates on a course, of which six or eight must be designated as upstream gates (International Canoe Federation, 2019). To negotiate an upstream gate the athlete must turn their boat to pass through the gate in an upstream direction, and the athlete uses the water currents to assist them in these manoeuvres.

There are currently two categories in canoe slalom: 1) C1 canoe where the athlete kneels in the boat and uses a single-bladed paddle; and 2) K1 kayak where the athlete sits in the boat and uses a two-bladed paddle with a blade at each end of the paddle shaft. For both categories a common aspect is that turning left into an upstream gate is typically done with the paddle blade on the left side of the boat, and turning right into an upstream is done with the paddle blade on the right. Additionally, modern canoe slalom courses are artificial, and provide multiple complex water features such as “stoppers” that need to be crossed to approach the upstream gates. However, due to the differences in the C1 and K1 paddle, the way that these turns and moves are achieved varies between the categories: for kayaking the double-bladed paddle naturally has a blade on each side of the boat, however, for the canoe category the athlete must move their paddle blade across the boat so that they can paddle on the different sides.

The nature of the single bladed paddle in C1 has thus led to the development of different types of paddle stroke for the canoe athletes. Canoe athletes hold the t-grip at the top of the paddle shaft with one hand, and place the other hand (the paddle hand) near the throat of the shaft just above the paddle blade. An onside-stroke is when the paddle hand used on its own side of the boat (e.g., right paddle hand used on right side of boat), whereas an off-side stroke is when the paddle hand and paddle blade is used on the other side of the boat (e.g., right paddle hand used on left side of boat). The athlete can keep their hands gripped on the same parts of the paddle when they transition between an on-side stroke on one side to an off-side stroke on the other side, and this is called a cross-transition. Alternatively, to transition between an on-side stroke on one side to an on-side stroke on the other side requires a switch transition where the athlete swaps the position of their hands between the t-grip and the throat. On-side and off-side strokes are asymmetrical to each other. These paddle strokes and transitions are illustrated elsewhere (Figure 1 in Tilden et al., 2021).

**FIGURE 1:**
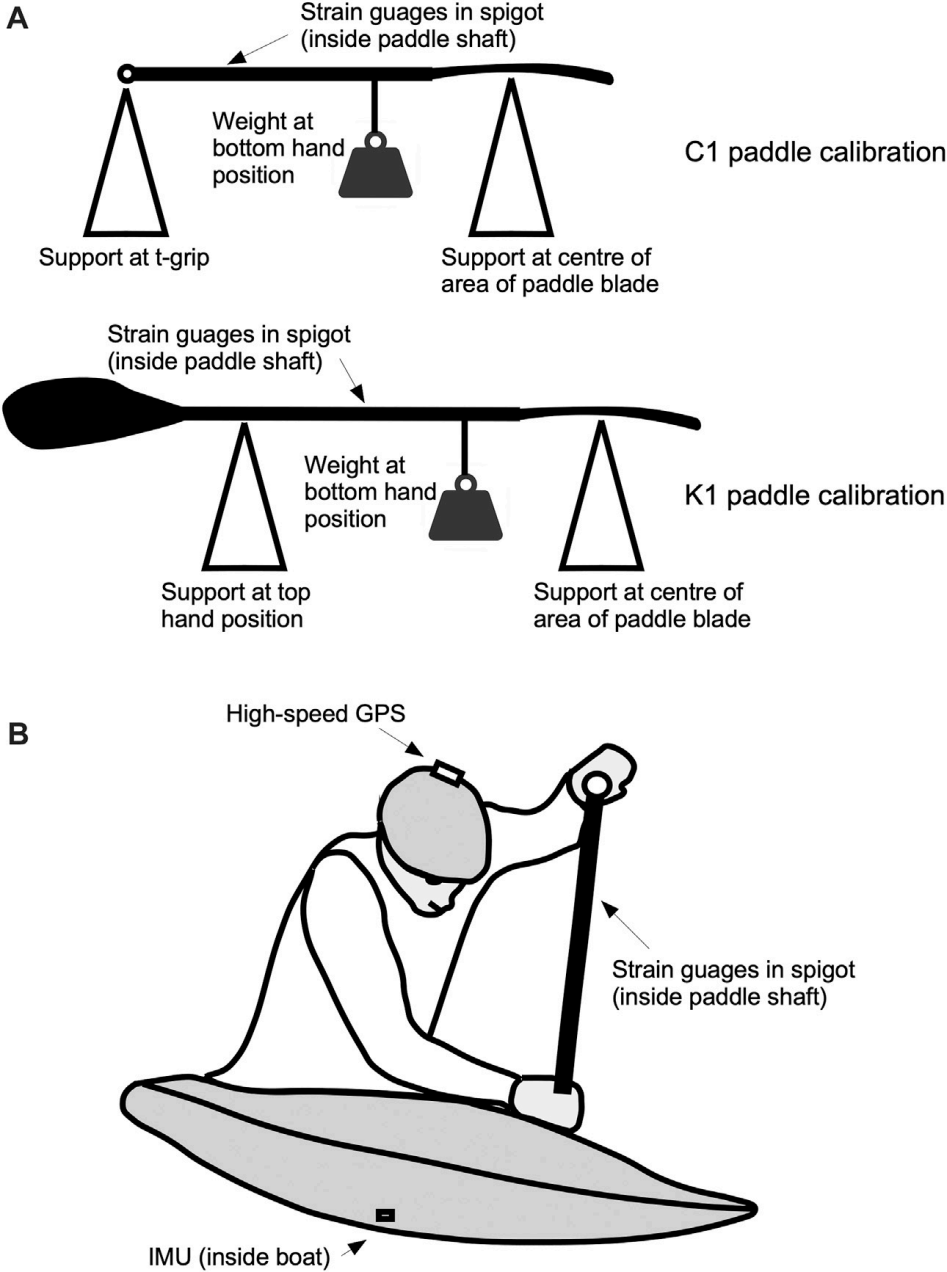
Methods and equipment for testing canoe slalom paddle forces. The C1 and K1 paddles were calibrated using a 3-point bending procedure **(A)**. High-speed GPS was placed on the paddler helmet, an IMU was taped inside the hull, and strain gauges measured the paddle forces **(B)**.

Male C1M athletes traditionally used cross transitions and off-side strokes to paddle on their non-dominant side. Female C1W athletes often use the switching transition, and this technique is being increasingly adopted by both younger and some elite male C1M athletes (Busta, 2020; Tilden et al., 2021). In order to transition the paddle blade across the boat for the up-stream gates, a canoe athlete will need to use at least six to eight cross-or switch transitions in a race, although at least 20 such transitions are typically made (Tilden et al., 2021). A survey of 33 international race runs between 2018 and 2020 showed that each cross transition is about 0.3 s faster than a switching transition (Tilden et al., 2021), and computer simulations predicted that if paddle strokes were of equal strength on the left and right sides of a boat, then the slower switching transition could lead to a small but significant increase in race time (Wakeling et al., 2022). Asymmetries in the strength of the strokes (in either C1 and K1 categories) can result in substantial differences in simulated race time (Wakeling et al., 2022) that are greater than the differences in simulated race time caused by cross- or switching-techniques in C1 (Wakeling et al., 2022).

Force imbalances have been shown for K1 paddlers (Macdermid et al., 2019; 2020). However, asymmetries in C1 paddle forces have not been reported, and specifically it is not known how paddle forces compare between on-side and off-side strokes, or between paddling on the dominant side (the side where a C1 athlete does most of their strokes) to the non-dominant side. Given that on-side and off-side C1 paddle strokes are asymmetrical to each other, and involve different body motions and likely muscle coordination, it is possible that the asymmetries in these C1 strokes are greater than the asymmetries between left and right strokes in K1 kayaking.

The purpose of this study was to provide empirical evidence whether there are asymmetries in the paddle force generated between on-side and off-side strokes for C1 slalom paddling, and to test the hypotheses that on-side C1 strokes would generate greater paddle forces than off-side and that on-side strokes would show similar paddle forces between the dominant and non-dominant sides due to these strokes being symmetrical. A set of kayak paddlers was additionally tested to determine how symmetrical a paddler could be, assuming that the symmetrical nature of the left and right K1 paddle strokes would lead to a symmetry in their left and right paddle forces.

## Methods

Twenty C1M and seven C1W canoe athletes and nine K1M and six C1W kayak athletes were tested, aged 20.2 ± 5.6 years (mean ± standard deviation). All athletes trained regularly and competed internationally in the testing season. The younger athletes were Developmental to National level (McKay et al., 2022) and the older athletes were Elite to World-class (McKay et al., 2022), including 17 who had won medals at canoe slalom European Championships, World Championships or Olympic Games. The athletes all provided oral consent to take part in the study, in accordance with requirements from the University Office of Research Ethics. Athlete testing occurred on flat-water portions of canoe slalom training sites at Roudnice na Labem, in Czechia and at Liptovský Mikuláš in Slovakia.

**FIGURE 2:**
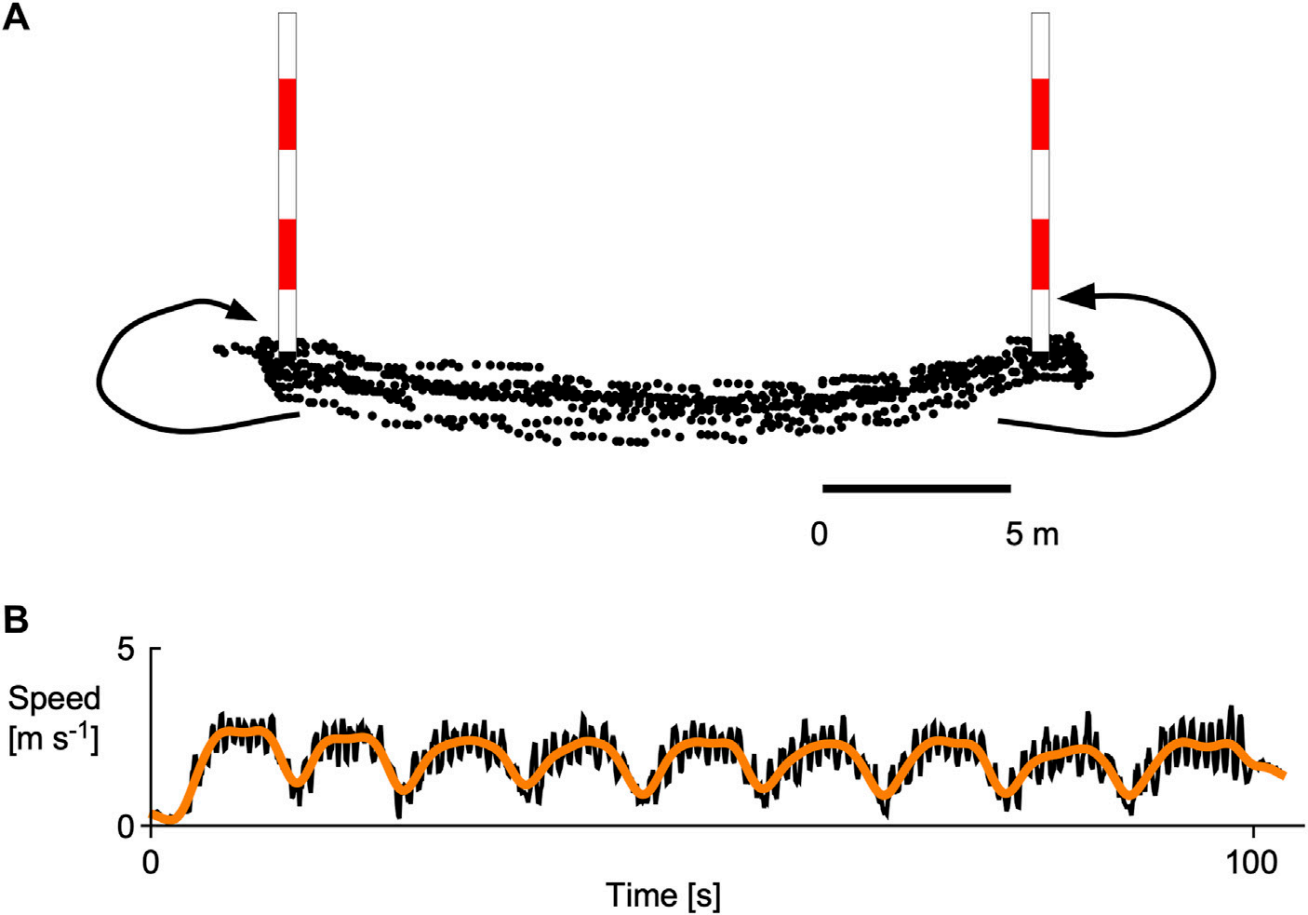
Testing regime for the canoe slalom athletes. The athletes paddled 9 lengths of a figure-of-8 course, turning in opposite directions around two slalom poles that were 20 m apart. The path of the athlete was tracked using high-speed GPS **(A)**. The athlete speed (low-pass filtered) was greatest during the lengths and slowed for the turns (orange line), and the individual strokes could be seen from the fluctuations in speed (black line) as shown in panel **(B)**. Data are shown for one C1 canoe athlete.

Athletes did an initial warm-up that included dry-land stretching and at least 10 min of paddling (technical strokes and getting accustomed to the testing equipment). Athletes then paddled two sets of figure-of-eight time trials around two slalom poles that were hanging above the water (Figure 2), athletes had a 10 min rest between trials. Athlete times were started when their torso passed the first pole, they paddled nine lengths (from one pole to the other), turning to the left around the second pole, and then to the right around the first pole, the time was stopped when their torso passed the second pole at the end of the nineth length. The C1 canoe athletes were asked to paddle one trial using switch transitions and onside strokes on both the left and right, and the second trial using cross-transitions and a combination of dominant-side onside strokes and non-dominant side offside strokes. A few athletes would only paddle using one of these strategies, and so they used the same strategy for both their trials. The K1 kayak athletes paddled using the same strategy for both trials.

Each athlete paddled their own boat, but they wore a high-speed satellite positioning system (10 Hz GPS and GLONAS systems, Glo 2, Garmin, Switzerland) strapped to their helmet to measure position and speed, and an inertial measurement unit (recording rate 25 Hz; MetamotionRL, Mbientlab, California, USA) taped inside their boat to measure boat orientation and acceleration. Paddle forces were measured using strain gauges that were embedded in a spigot that was secured in the paddle shafts between the positions of the top and bottom hands (recording rate 100 Hz; Canoe Power Meter second Gen., One Giant Leap, New Zealand), and they were filmed using 60 Hz video. The paddles were equipped with medium-sized blades (Revolution for C1 and Hunter for K1, both G’Power, Poland). The paddles were previously calibrated using a three-point bending protocol (similar to Aitken and Neal, 1992) where the horizontal paddle shaft was supported at the centre of area of the paddle blade, and at a range of top-hand positions (t-grip for C1, or hand position for K1). A series of six static weights from 7 to 41 kg were hung from the position of the bottom paddle hand to apply a vertical bending force to the shaft. A linear model was made to calibrate the strain in the paddle shaft to the shaft lengths and the hand positions on the paddle. For the C1 paddle, the calibrations yielded *r* ^2^ > 0.99 and a root-mean-square error (RMS) of 1.9 N over a 0–256 N range of paddle forces. For the K1 paddles, the calibrations yielded *r* ^2^ > 0.99 and RMS errors of 2.2 N for the left and 3.2 N for the right blade over a 0–220 N range of paddle forces.

Synchronization of the data was achieved using cross-correlation of the paddle forces with the boat accelerations (either directly from the IMU, or from second derivative of the GPS positions). The video recordings were used to classify each stroke type. All data processing was conducted in custom software (Wolfram Research, Inc., Mathematica version 13, Champaign, IL).

Post-processing divided the data into individual paddle strokes that were classified into left or right side and on-side or offside strokes using the video. The turning strokes around the pole were excluded, as identified from the video, and so only the forward strokes were used for further analysis. The dominant side for each C1 athlete was taken as the side that the majority of on-side strokes were used. Kayak athletes have one control hand that grips the shaft securely, and this could be either the left or right hand, depending on the athlete. There were a mix of “left” and “right” handed kayakers in this study. However, because a similar number of left and right strokes are used for forward K1 paddling, the dominant side for the K1 athletes was taken as the side that generated the greatest mean paddle forces. For each paddle stroke the peak force was determined as well as the impulse, which was the time-integral of the paddle force over the entire stroke cycle. The left and right strokes were analyzed separately for the K1 data.

### Statistics

The peak paddle forces were evaluated by analyses of covariance (ANCOVA) using the SPSS version 27 statistical package. Stroke type and athlete sex were included as factors, and athletes were distinguished by either their age or mass, that were used as co-variates. The peak paddle forces increased with athlete mass for the canoe athletes throughout the range of masses tested, 47–89 kg, however the female C1W athletes were all in the lower range of under 70 kg. For this reason, the effect of stroke-type and sex as factors on the peak paddle forces was estimated for both the entire data set, and also for the subset of athletes that were under 70 kg, in an attempt to provide a more balanced comparison between the male and female athletes. Similarly, the peak paddle forces increased with athlete age for the canoe athletes up to about 23 years, however, the female C1W athletes were all in the lower range of under 24 years. For this reason, the effect of stroke-type and sex as factors on the peak paddle forces was estimated for both the entire data set, and also for the subset of athletes that were under U23 (less that 24 years old), in an attempt to provide a more balanced comparison between the male and female athletes. The majority (75%) of the force distributions for peak paddle forces for each athlete-stroke type combination were normally distributed (Kolomogorov-Smirnov test; *p* < 0.05), and the large number of combinations (*N* = 96) allow statistical inferences to be made from these ANCOVAs due to the central limit theorem (Kwak and Kim, 2017). Effects were deemed to be statistically significant at the *p* < 0.05 level. The peak paddle forces are described by their estimated marginal means (with standard error of the mean) that emerge from these ANCOVAs.

## Results

**FIGURE 3:**
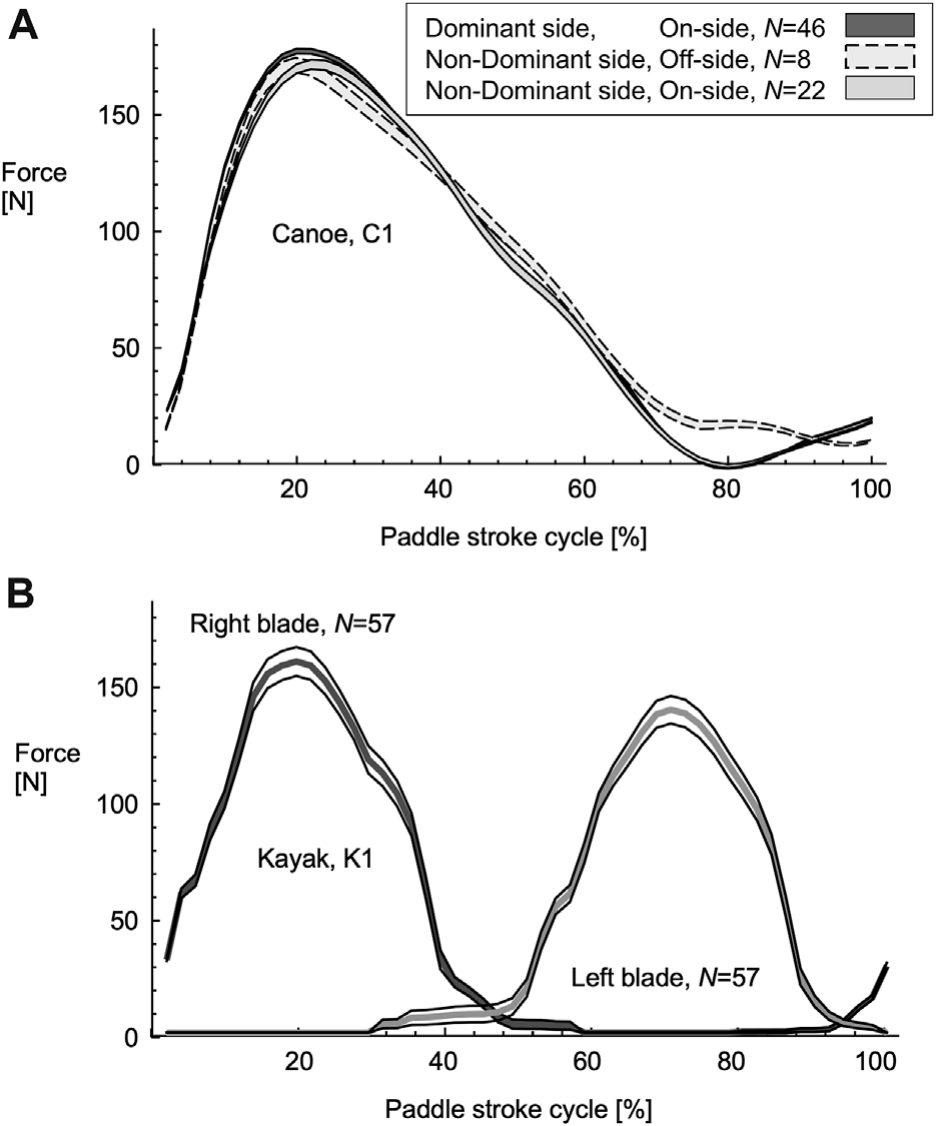
Paddle forces for canoe, C1 **(A)** and kayak, K1 athletes **(B)**. The paddle forces are shown for the straight paddling section of the figure-of-8 course. Data are shown for one C1M canoe athlete and one K1W Kayak athlete: the lines show the mean ± standard error of the mean. The different stroke types are distinguished for the canoe, and the right and left strokes distinguished for the kayak athlete.

Paddle forces were analysed for a total of 4,564 forward strokes for the canoe and 6,276 forward strokes for the kayak athletes. For the canoe strokes, 72% were dominant-side onside strokes, 16% were non-dominant side onside strokes, 11% were non-dominant side offside strokes, and less than 1% were dominant-side offside strokes. These dominant-side offside strokes were not analysed further. The C1 paddle forces peaked in the first half of the pull-phase of the stroke (20.94% ± 0.17% of stroke cycle: example for one athlete in Figure 3A). For the kayakers, the side with the greatest peak forces was classified as the dominant side for each athlete, and they paddled equal numbers of strokes on their dominant and non-dominant sides: 3,138 strokes for each side. The K1 paddle forces peaked later in the pull-phase of the stroke (28.41% ± 0.22% of stroke cycle: example for one athlete in Figure 3B).

The estimated marginal mean for the peak paddle force for the C1 was 177.0 ± 1.5 N and for the K1 was 104.2 ± 0.8 N. However, it is the impulse (area under the force-time graph) that is important for overcoming the effects of drag over the whole stroke cycle. The impulse was similar for the two types of paddling: the impulse for a C1 stroke cycle was 56.4 ± 2.4 N s and the impulse for the K1 was 57.1 ± 0.5 N s (left and right sides combined).

**FIGURE 4:**
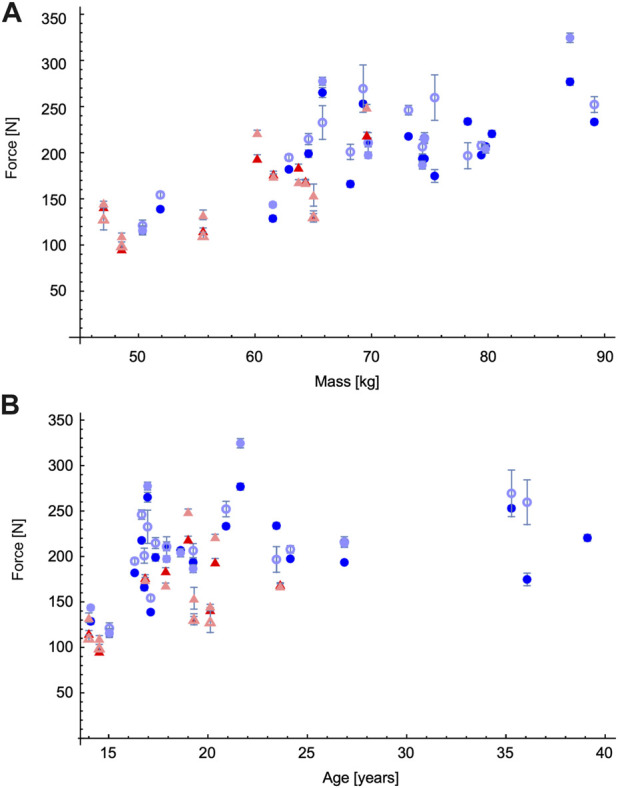
Peak forces for canoe, C1 athletes, shown in relation to athlete mass **(A)** and athlete age **(B)**. Female C1W athletes are shown in red, and male C1M in blue. Stroke types are distinguished by colour: dominant-side on-side with dark colours, non-dominant side off-side strokes with open symbols, and non-dominant side on-side with light colours: note the dominant side is the side that the majority of on-side strokes are used. Each point shows the mean ± standard error of the mean for one athlete.

The peak paddle forces increased with athlete mass for the canoe athletes throughout the range of masses tested, 47–89 kg (Figure 4A), however the female C1W athletes were all in the lower range of under 70 kg. For this reason, the effect of stroke-type and sex as factors on the peak paddle forces was estimated for both the entire data set, and also for the subset of athletes that were under 70 kg, in an attempt to provide a more balanced comparison between the male and female athletes. The peak paddle forces increased with athlete age for the canoe athletes up to about 23 years (Figure 4B), additionally, the female C1W athletes were all in the lower range of under 24 years. For this reason, the effect of stroke-type and sex as factors on the peak paddle forces was estimated for both the entire data set, and also for the subset of athletes that were under U23 (less that 24 years old), in an attempt to provide a more balanced comparison between the male and female athletes. All these ANCOVAs showed significant main effects of the stroke type (*p* < 0.001), athlete sex (*p* < 0.001) and the interaction between stroke type and sex (*p* < 0.001) on the peak paddle force.

**FIGURE 5:**
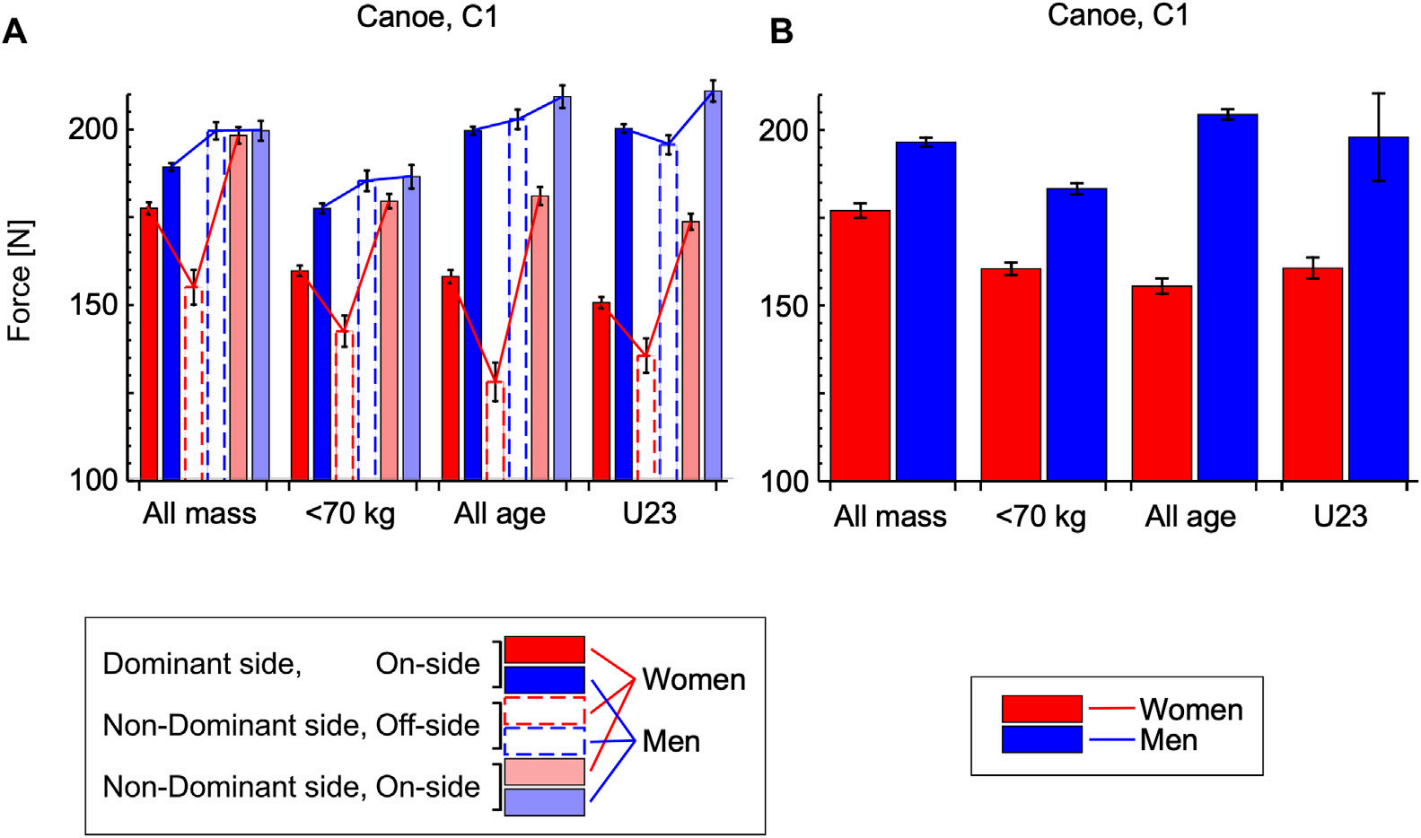
Estimated marginal means for the canoe, C1 peak paddle forces. Data are shown for four separate analyses: using mass as a covariate for (i) all athletes [evaluated for a mass of 67.6 kg], and (ii) for athletes <70 kg [evaluated for a mass of 61.0 kg]; and using age as a covariate for (iii) all athletes [evaluated for an age of 20.9 years], and for (iv) U23 athletes [evaluated for an age of 18.2 years]. Female C1W athletes are shown in red, and male C1M in blue. Stroke types are distinguished by colour: dominant-side on-side with dark colours, non-dominant side off-side strokes with open bars and dashed surround, and non-dominant side on-side with light colours: note the dominant side is the side that the majority of on-side strokes are used. Each bar shows the estimated marginal mean ± standard error of that mean. Graphs show the estimated marginal means for stroke type by sex **(A)** and athlete sex **(B)**. ANCOVAs showed significant main effects of the stroke type, athlete sex and the interaction between stroke type and sex on the peak paddle force.

The estimated marginal means for the C1 peak paddle forces distinguished by stroke type and sex are shown in Figures 5A,B, respectively and Table 1. The onside strokes on the non-dominant side had greater peak paddle forces than the onside strokes on the dominant side, with the difference being 4.9%–5.5% for the male C1M athletes and 11.8%–15.4% for the C1W athletes. The offside strokes (on the non-dominant side) had similar peak paddle forces to the onside strokes on the non-dominant side for the C1M, and the range of differences in the estimated marginal means was 0.0% to −7.3%. By contrast, the offside strokes for the C1W had peak paddle forces less than the onside strokes for the non-dominant side, with the range of differences in the marginal means being −20.8% to −29.5%. When the data were mass or age-adjusted using these ANCOVAs, the estimated marginal means showed that the peak paddle forces for female C1W athletes were −9.9% to −23.8% less than the peak paddle forces for the male C1M athletes, and this effect was statistically significant (*p* < 0.001).

**TABLE 1:** Estimated marginal means and 95% confidence intervals for the canoe, C1 peak paddle forces [N]. Details for the four analyses are described in Fig. 5.

Covariate			Mass				Age			
Stroke type		Sex	All mass		<70 kg		All age		U23	
			Mean ± *s*.e.m	95% CI	Mean ± *s*.e.m	95% CI	Mean ± *s*.e.m	95% CI	Mean ± *s*.e.m	95% CI
		Factors: Stroke type by Sex								
On-side	Dominant side	F	177.4 ± 1.7	174.0–180.8	159.5 ± 1.5	156.6–162.4	157.9 ± 1.8	154.3–161.5	150.4 ± 1.6	147.2–153.5
		M	189.3 ± 1.1	187.2–191.4	177.4 ± 1.4	174.6–180.1	199.7 ± 1.2	197.4–202.0	200.4 ± 1.2	197.9–202.8
Off-side	Non-Dominant side	F	154.8 ± 5.0	145.1–164.5	142.2 ± 4.5	133.4–151.0	127.6 ± 5.5	116.9–138.4	135.2 ± 4.9	125.5–144.8
		M	199.7 ± 2.5	194.8–204.5	185.3 ± 2.9	179.6–191.1	203.0 ± 2.8	197.5–208.4	195.7 ± 2.7	190.3–201.0
On-side	Non-Dominant side	F	198.4 ± 2.4	193.7–203.0	179.5 ± 2.1	175.4–183.6	181.1 ± 2.6	175.9–186.0	173.6 ± 2.3	169.1–178.1
		M	199.7 ± 2.9	194.1–205.2	186.5 ± 3.4	179.9–193.1	209.5 ± 3.2	203.2–215.8	211.1 ± 3.0	205.1–217.1
		Factor: Sex								
		F	176.9 ± 2.0	172.9–180.8	160.4 ± 1.7	157.0–163.8	155.5 ± 2.1	151.3–159.7	160.6 ± 3.0	154.7–166.4
		M	196.2 ± 1.3	193.4–198.8	183.1 ± 1.6	180.0–186.2	204.0 ± 1.5	159.7–206.9	197.6 ± 12.4	173.4–221.9

**FIGURE 6:**
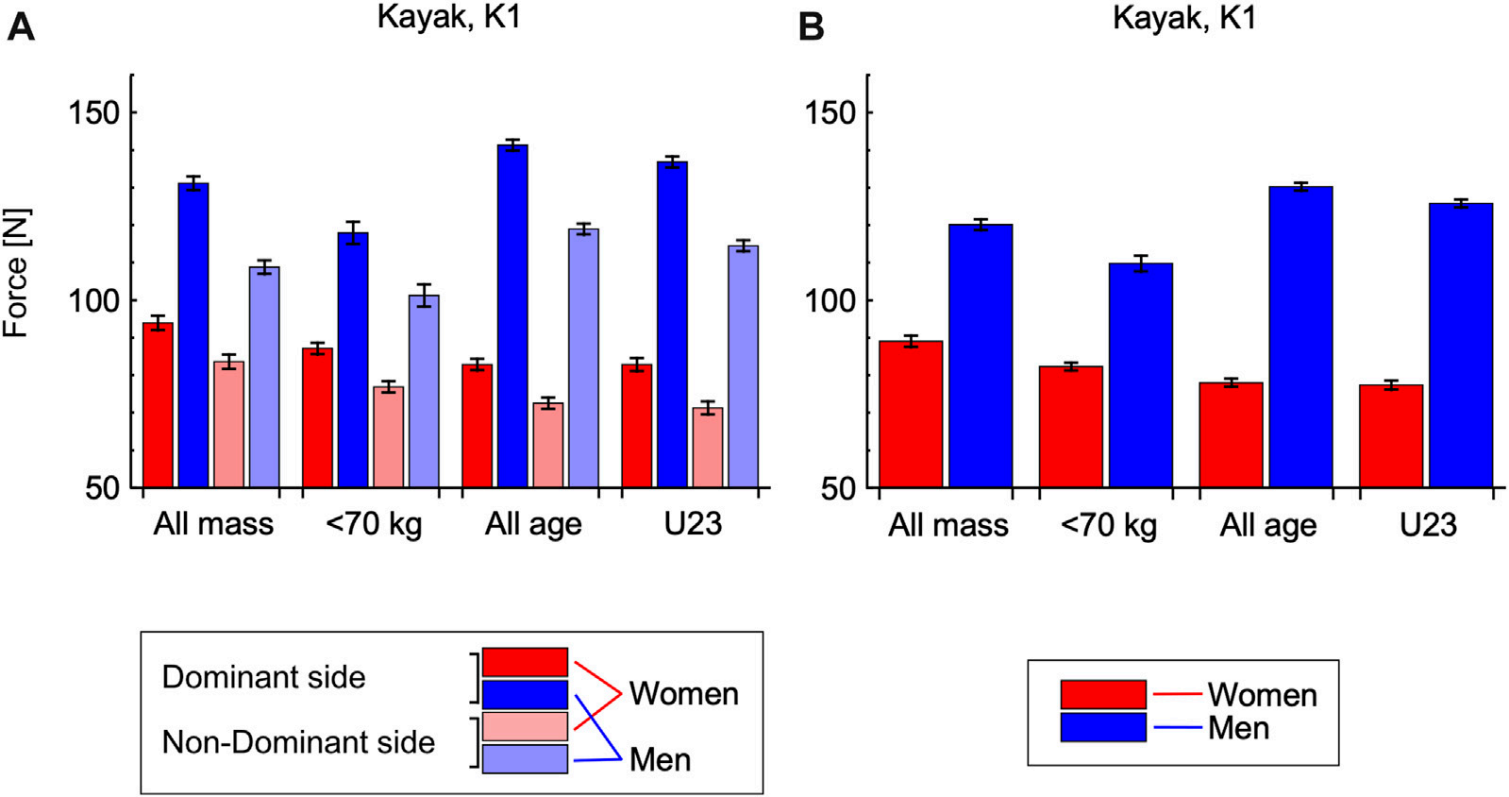
Estimated marginal means for the kayak, K1 peak paddle forces. Data are shown for four separate analyses: using mass as a covariate for (i) all athletes [evaluated for a mass of 65.4 kg], and (ii) for athletes <70 kg [evaluated for a mass of 59.0 kg]; and using age as a covariate for (iii) all athletes [evaluated for an age of 18.8 years], and for (iv) U23 athletes [evaluated for an age of 18.3 years]. Female K1W athletes are shown in red, and male K1M in blue. Stroke types are distinguished by colour: dominant-side with dark colours and non-dominant with light colours: note the dominant side for kayaking is the side that the athlete generates the greatest peak paddle forces. Each bar shows the estimated marginal mean ± standard error of that mean. Graphs show the estimated marginal means for stroke type by sex **(A)** and athlete sex **(B)**. ANCOVAs showed significant main effects of the stroke side and athlete sex on the peak paddle force.

The estimated marginal means for the K1 peak paddle forces distinguished by stroke side and sex are shown in Figures 6A,B, respectively and Table 2. The strokes on the non-dominant side had statistically significantly lower peak paddle forces than the strokes on the dominant side (*p* < 0.001), with the difference being −14.2% to −17.1% for the male K1M athletes and −11.1 to −14.4 for the K1W athletes. When the data were mass or age-adjusted using these ANCOVAs, the estimated marginal means showed that the peak paddle forces for female K1W athletes were −25.2% to −40.4% less than the peak paddle forces for the male K1M athletes, and this effect was statistically significant (*p* < 0.001).

**TABLE 2:** Estimated marginal means and 95% confidence intervals for the kayak, K1 peak paddle forces [N]. Details for the four analyses are described in Fig. 6.

Covariate		Mass				Age			
Stroke type	Sex	All mass		<70 kg		All age		U23	
		Mean ± *s*.e.m	95% CI	Mean ± *s*.e.m	95% CI	Mean ± *s*.e.m	95% CI	Mean ± *s*.e.m	95% CI
	Factors: Stroke type by Sex								
Dominant side	F	94.1 ± 1.9	90.3–97.9	87.3 ± 1.5	84.4–90.3	83.0 ± 1.5	80.0–85.9	83.0 ± 1.7	79.6–86.3
	M	131.5 ± 1.8	128.0–135.1	118.2 ± 3.0	112.4–124.0	141.7 ± 1.5	138.9–144.6	137.2 ± 1.5	134.3–140.1
Non-Dominant side	F	83.8 ± 1.9	80.0–87.5	77.0 ± 1.5	74.0–79.9	72.6 ± 1.5	70.0–75.6	71.4 ± 1.7	68.0–74.7
	M	109.1 ± 1.8	105.5–112.6	101.5 ± 3.0	95.7–107.3	119.3 ± 1.5	116.4–122.1	114.8 ± 1.5	111.9–117.7
	Factor: Sex								
	F	88.9 ± 1.5	86.0–91.9	82.2 ± 1.1	80.0–84.2	77.8 ± 1.1	75.7–79.9	77.2 ± 1.2	74.8–80.0
	M	120.3 ± 1.4	117.5–123.1	109.9 ± 2.1	105.7–114.0	130.5 ± 1.0	128.5–132.5	126.0 ± 1.0	124.0–128.0

## Discussion

The C1 paddle forces were in general higher than the K1 paddle forces. However, the kayak athletes generate paddle force during more of the stroke cycle (with either the left or the right blade of the paddle), whereas the canoe athletes have a more prolonged transition period when the single paddle blade moves from the end of one stroke to the start of the next. The resulting impulse from the complete stroke cycles were similar for the two categories of boat. This study reports the paddle forces because these are the propulsive forces for the athlete. The paddle forces declined as the athletes fatigued during these 90 s tests (reduction in peak force of −20.6% ± 1.4% for C1 and -29.7% ± 2.5% for K1: similar to the decline in forces reported during simulated flat-water races: Macdermid et al., 2019): the mean of the peak paddle force for the K1 athletes across their entire runs was 104 N, and their impulse was 57 N s per complete stroke cycle. There are no equivalent paddle force data for slalom paddling in the literature. However, by comparison elite adult sprint paddlers doing a 200 m course at 60 strokes per minute (similar to the cadence in slalom paddling) generate 126–225 N of peak force (Gomes et al., 2015) and the peak paddle forces for maximal paddling of one length of a pool for canoe polo athletes (who paddle kayaks: Löppönen et al., 2022) is 125 N. With one hand pushing and one hand pulling during a canoe or kayak stroke, the peak paddle force will be the difference in force between the two hands and thus will be less than the peak hand force. Indeed, the peak hand forces across eight K1 slalom paddlers was reported at 184 N, but they achieved an impulse of 62 N s that was similar to the impulses recorded in our study (Macdermid et al., 2019).

It has been suggested that the ideal profile of the paddle forces would likely be rectangular for flat-water sprint paddling (Michael et al., 2009; Gomes et al., 2015). The profiles for the paddle forces in this study were more skewed and triangular than rectangular, seen by the ratio of the mean pull phase force to the peak force (51.0% ± 0.01% for C1 and 52.6% ± 0.01% for K1): these ratios are similar to the profiles reported for sprint kayaking at a low cadence of 60 strokes per minute (53.3%: Gomes et al., 2015). It should be noted that the C1 paddle forces peaked at an earlier time than the K1 forces (21% vs. 28% stroke cycle; also see Wakeling et al., 2022), and such skewness in the force profile leads to faster simulated race times for a given impulse of the paddle stroke (Wakeling et al., 2022).

For the C1 canoe athletes the paddle forces for the on-side strokes on the non-dominant side were slightly higher than the paddle force for the on-side strokes on the dominant side (Figure 5). This may have been due to the reduced number of on-side strokes on the non-dominant side (16%) compared to on-side strokes on the dominant side (72%) leading to less muscle fatigue for these on-side strokes. The on-side stroke asymmetries for the male C1M athletes were less than for the K1M athletes (4.9%–5.5% compared to 14.2%–17.1%), whereas the on-side stroke asymmetries were similar between the female C1W and K1W athletes (11.8%–15.4% compared to 11.1%–14.4%).

A previous computer model has simulated the race times for C1 slalom paddling (Wakeling et al., 2022). The model used forces from straight paddling on flat water or an indoor ergometer and showed that using fewer C1 switch transitions, that take longer than cross-transitions, could lead to faster race times, and it only takes 3 fewer switch transitions to reach the smallest worthwhile performance enhancement (of 0.4 s) that can increase the prospects of winning a medal (Hopkins et al., 1999). However, the model also predicted that a more substantial factor in race times could be an asymmetry in paddle force between the dominant and non-dominant sides. Indeed, the results from the simulations (Wakeling et al., 2022) showed that it would only take 0.5% in stroke asymmetry to reach this smallest worthwhile performance enhancement. However, the actual forces in canoe slalom are more complex due to the nature of the white water and the additional types of turning stroke (K1 forces: Macdermid et al., 2020; Macdermid and Olazabal, 2022). The data from this study (Figures 5, 6) show that substantial asymmetries exist in the forces for the forward strokes between the dominant and non-dominant sides for canoe slalom athletes that are sufficient to result slower race times. Thus, one way to enhance athlete performance may be to develop better symmetry in strength in paddle forces between the dominant and non-dominant sides.

The male C1M canoe athletes generated paddle forces that were similar for the on-side and off-side strokes on the non-dominant side (Figure 5). Again, drawing inference from the computer simulations (Wakeling et al., 2022), these results suggest that there would be little difference in race time for male athletes paddling on-side or off-side strokes on their non-dominant side and this is particularly the case for the downstream sections of the course that the computer simulations most closely mimic. This is not to say that C1M paddlers should not use switch transitions because switching to achieve on-side strokes to negotiate complex features on the approach to up-stream gates may become increasingly important as modern course designs become more technical. Additionally, switching allows for a more balanced training across the body, minimises repetitive unilateral muscle contractions and joint movements, may minimise muscle fatigue and may enhance an athlete’s stability or efficiency for challenging moves (Busta, 2020). However, multiple and unnecessary switching transitions to achieve on-side strokes on both the dominant and non-dominant side could lead to longer race times (Wakeling et al., 2022).

By contrast, the female C1W canoe athletes generated paddle forces that were significantly less for their off-side than for their on-side strokes on the non-dominant side (Figure 5): their off-side non-dominant side stroke forces were 21%–30% less than their on-side strokes on their non-dominant side and 9%–19% less than the on-side strokes on the dominant side. These data (in conjunction with the computer simulation: Wakeling et al., 2022) suggest that the C1W athletes would likely result in faster race times by minimizing the number of off-side strokes. This is the case even for the downstream sections of the course due to the reduction in paddle force for their off-side strokes. Thus, the data indicate that C1W athletes would benefit from using switch transitions most of the time when moving the paddle blade from one-side of the boat to the other.

The paddle forces from both the C1 and K1 athletes were lower for the female athletes than the male athletes (Figures 5, 6), and this was even when the data were adjusted for the fact that the female athletes were lighter than their male counterparts, and that no senior female athletes were tested that would have potentially more experience. It is interesting to note that the regulations for slalom boat size and weight, and for course design are the same for both male and female competitions (International Canoe Federation, 2019). However, these data suggest that the optimal boat and course design may be different for male and female athletes because of their inherent differences in strength and this may need to be a consideration for the evolution of the sport in future.

This study shows that there are sex-based differences in paddle forces for C1 canoe slalom athletes, and in particular there are sex-based differences in the paddle forces for the strokes on the non-dominant side for the female paddlers that are not apparent for the male paddlers. These differences may explain the higher proportion of on-side strokes on the non-dominant side, and higher number of switch transitions used by the female paddlers (Tilden et al., 2021). Male athletes traditionally used cross transitions and off-side strokes to paddle on their non-dominant side (Busta, 2020). Despite an increasing number of younger male athletes being introduced to the switching technique, and it being used by C1M athletes in international competitions since 2014 (Busta, 2020), C1M paddlers still do not use switching transitions as much as C1W (Tilden et al., 2021). We infer from this study that there may be a biomechanical reason for this sex-based difference in C1 stroke choice: this needs to be considered for optimal technique development and race performance and warrants further investigation.

### Limitations

This study used one K1 paddle and one C1 paddle for all athletes, and so the paddles did not handle the same as the athletes’ own equipment: the paddles were heavier due to the instrumentation (the C1 paddle was 33 g heavier than an equivalent non-instrumented paddle) and an intermediate size paddle blade was used for all athletes. Thus, each athlete may not have paddled at their optimal level given their unfamiliarity with the equipment. However, the main results of the study are the relative differences in paddle force between the different types of paddle stroke and not the absolute performance of individual athletes, and thus be relatively insensitive to the choice of equipment used. Additionally, the data analyzed in this study were from straight strokes on a flat-water course, and so the conclusions will be most relevant to flatter-water scenarios. As the water difficulty increases, the water dynamics (Vajda and Piatrikova, 2022), and the technical skill and experience of the athlete (Busta, 2020) become increasingly important in determining race performance.

## Practical implications

The following are recommendations about stroke choice and stroke technique for canoe slalom athletes and their coaches.

• Both K1 and C1 paddlers have asymmetries in paddle force that are large enough to impact their performance. Developing a smooth technique to balance paddle forces between the left and right sides will improve performance.
• C1W generate reduced paddle forces with their off-side strokes. On-side strokes on both left and right sides would be a choice to maximize speed.
• C1M generate substantial paddle forces with their off-side strokes. The choice of on-side or off-side strokes should be determined by the skill of the athlete and the technical moves that need to be achieved.
• Excessive switching transitions for either C1W and C1M result in longer simulated race times. Developing the boat control necessary to reduce the number of switching transitions would lead to increased performance.

## Data Availability

The original contributions presented in the study are included in the article, further inquiries can be directed to the corresponding author.

## References

[B1] Aitken D. A. Neal R. J. (1992). An on-water analysis system for quantifying stroke force characteristics during kayak events. Int. J. Sport Biomech. 8, 165–173. 10.1123/ijsb.8.2.165

[B2] Busta J. (2020). Za úspěchem ve vlnách. Euromedia. (in Czech).

[B3] Gomes B. B. Ramos N. V. Conceição A. V. Ross F. Sanders R. H. Vaz M. A. (2015). Paddling force profiles at different stroke rates in elite sprint kayaking. J. Appl. Biomech. 31, 258–263. 10.1123/jab.2014-0114 25838207

[B4] Hopkins W. G. Hawley J. A. Burke L. M. (1999). Design and analysis of research on sport performance enhancement. Med. Sci. Sports Exerc. 31, 472–485. 10.1097/00005768-199903000-00018 10188754

[B5] International Canoe Federation (2019). Rules canoe slalom 2019. Available at: https://www.canoeicf.com/sites/default/files/rules_canoe_slalom_2019.pdf (Accessed October 12, 2020).

[B6] Kwak S. G. Kim J. H. (2017). Central limit theorem: the cornerstone of modern statistics. Korean J. Anesthesiol. 70, 144–156. 10.4097/kjae.2017.70.2.144 28367284 PMC5370305

[B7] Löppönen A. Tomi V. Marko H. Vesa L. (2022). The effect of paddle stroke variables measured by Trainesense SmartPaddle® on the velocity of the kayak. Sensors 22, 938. 10.3390/s22030938 35161684 PMC8840261

[B8] Macdermid P. W. Gilbert C. Jayes J. (2020). Using a kayak paddle power-meter in the sport of whitewater slalom. J. Hum. Sport Exerc. 15 (1), 105–118. 10.14198/jhse.2020.151.10

[B9] Macdermid P. W. Olazabal T. (2022). The relationship between stroke metrics, work rate and performance in slalom kayakers. Biomechanics 2 (1), 31–43. 10.3390/biomechanics2010005

[B10] Macdermid P. W. Osborne A. Stannard S. R. (2019). Mechanical work and physiological responses to simulated flat water slalom kayaking. Front. Physiol. 10, 260. 10.3389/fphys.2019.00260 30949065 PMC6436605

[B11] McKay A. K. Stellingwerff T. Smith E. S. Martin D. T. Mujika I. Goosey-Tolfrey V. L. (2022). Defining training and performance caliber: a participant classification framework. Int. J. Sports Physiol. Perform. 17, 317–331. 10.1123/ijspp.2021-0451 34965513

[B12] Michael J. S. Smith R. Rooney K. B. (2009). Determinants of kayak paddling performance. Sports Biomech. 8, 167–179. 10.1080/14763140902745019 19705767

[B13] Tilden M. L. Oberoi A. R. Wakeling J. M. (2021). Canoe slalom C1 stroke technique during international competitions. Sports Biomech. 2021, 1–12. 10.1080/14763141.2021.1942968 34154513

[B14] Vajda M. Piatrikova E. (2022). Relationship between flat-water tests and canoe slalom performance on 4 different grades of water terrain difficulty. Int. J. Sports Physiol. Perform. 17, 185–194. 10.1123/ijspp.2021-0115 34611060

[B15] Wakeling J. M. Pratt J. S. Smiešková S. (2022). Stroke technique in C1 canoe slalom: a simulation study. Sports Biomech., 1–11. 10.1080/14763141.2022.2088401 35726479

